# Case Report: Clinical Analysis of Fulminant *Mycoplasma pneumoniae* Pneumonia in Children

**DOI:** 10.3389/fped.2021.741663

**Published:** 2021-12-09

**Authors:** Tongqiang Zhang, Chunjiao Han, Wei Guo, Jing Ning, Chunquan Cai, Yongsheng Xu

**Affiliations:** ^1^Department of Pulmonology, Tianjin Children's Hospital/Tianjin University Children's Hospital, Tianjin, China; ^2^Clinical School of Paediatrics, Tianjin Medical University, Tianjin, China; ^3^Institute of Pediatrics, Tianjin Children's Hospital/Tianjin University Children's Hospital, Tianjin, China

**Keywords:** fulminant *Mycoplasma pneumoniae* pneumonia, children, clinical diagnosis, fiberoptic bronchoscopy, treatment

## Abstract

Fulminant *Mycoplasma pneumoniae* pneumonia (FMPP) accounts for 0.5–2% of all MPP cases, which is considered as MPP combined with severe complications such as hypoxemia, acute respiratory distress syndrome, or acute respiratory failure. It primarily affects young adults with no underlying disease. Although some studies have proved the severity of FMPP, the details about clinical diagnosis and treatment of FMPP in children have been rarely reported. In this case study, we described three cases who suffered from FMPP. These children not only developed acute lung injury and multiple organ involvement within 7 days of treatment, but were also found plastic bronchitis by bronchoscopy. Finally, all the patients were treated successfully with azithromycin, glucocorticoid, and bronchoscopy lavage. We conclude that this case study would contribute to raise awareness with respect to FMPP, which may occur at a younger age with faster disease progression and common extrapulmonary manifestations. It also reinforces the importance of early identification and prompt intervention to save life of children and reduces sequelae. Further studies are needed about mechanism of FMPP.

## Introduction

*Mycoplasma pneumoniae* (MP), an important pathogenic microorganism in clinical practice, is a common cause of respiratory tract infections in school-aged children (1). MP pneumonia (MPP) is an acute infection caused by MP. Although MPP is considered self-limited and can be resolved spontaneously without specific treatment, 0.5–2% of MPP cases is known to present fulminant MPP (FMPP) with severe complications such as acute respiratory distress syndrome and respiratory failure, which primarily affects young adults with no underlying disease (2, 3). Several studies have proved resultant respiratory failure and death in patients with FMPP (2, 4, 5); however, the details about clinical diagnosis and treatment of FMPP in children have been rarely reported.

In this case study, we discussed the diagnosis and treatment in three children with FMPP.

## Case Description

### Case 1

**Figure 1:**
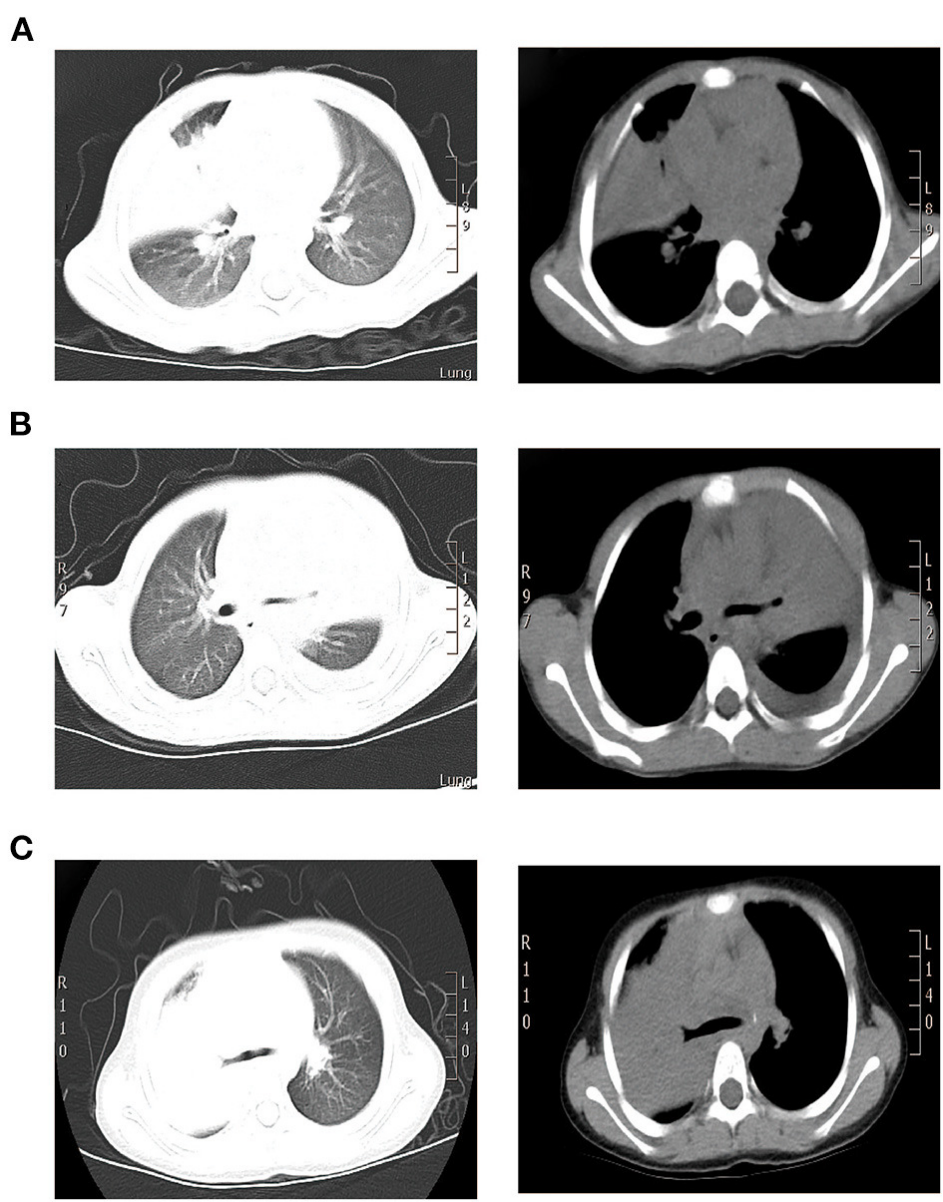
CT scan findings of three cases. **(A)** Pulmonary CT results of case 1 suggested consolidation with atelectasis in the middle lobe of right lung. **(B)** Pulmonary CT of case 2 suggested inflammatory consolidation in the upper and middle lobes of left lung with left pleural effusion. **(C)** Pulmonary CT of case 3 suggested substantial pulmonary consolidation in the upper and middle lobes of right lung accompanied by significant effusion (Left: lung window image; Right: mediastinal window image).

A 1.9-year-old girl was referred to our hospital on March, 2016 due to 4-day fever and cough, with no pertinent past medical history. Physical examination revealed temperature 37.4°C, heart rate 120 beats/min, respiration 50 times/min, blood pressure 90/60 mm Hg, and transcutaneous oxygen saturation 92% without oxygen administration, fatigue, and depressions in suprasternal fossa, supraclavicular fossa, and intercostal space. She developed hypoxemia, so the reservoir mask of 6 l/min was utilized for ventilatory support. The right lung showed diminished breath sounds. Cardiovascular, nervous system, extremities, antinuclear antibodies (ANAs), and extractable nuclear antigens (ENAs) examinations were normal. Routine blood tests showed the following results: hemoglobin (Hb) 118 g/l, white blood cell (WBC) 7.83 × 10^9^/L, neutrophils (N) 53.3%, lymphocytes (L) 40.4%, and C-reactive protein (CRP) 156 mg/l. Arterial blood gas analysis revealed a pH of 7.48, partial pressure of carbon dioxide in artery (PaCO_2_) of 42 mm Hg, partial pressure of oxygen in artery (PaO_2_) of 50 mm Hg, base excess (BE) of 6.9 mmol/l, and oxygenation index of 238. Pulmonary CT suggested consolidation with atelectasis in the middle lobe of right lung (Figure 1A).

**Figure 2:**
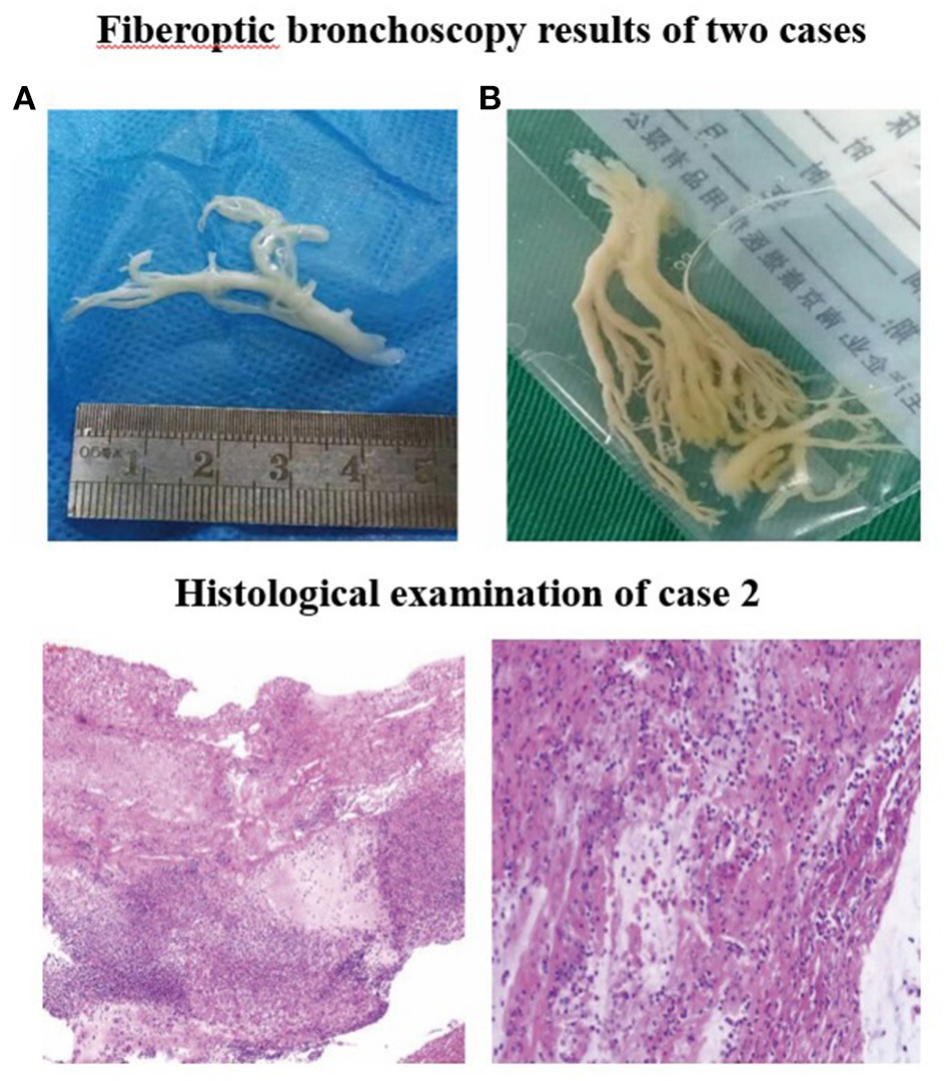
Fiberoptic bronchoscopy results of two cases. Fiberoptic bronchoscopy revealed the formation of plastic bronchial tree in the airway of **(A)** case 1 and **(B)** case 2, indicating plastic bronchitis. Histological examination of case 2. Pathologic findings indicated that case 2 was confirmed to have plastic bronchitis.

Electrocardiogram indicated sinus rhythm with blunt T wave of part of the lead, visible in double peak. The patient received cephalothin for anti-infection at admission. On 2nd day, due to pneumonia complicated with atelectasis in her CT scan, the first fiberoptic bronchoscopy (FB) was used to relieve atelectasis and obtain respiratory samples for bacteriologic, cytologic, and histologic detection. On 3rd day, the titer of MP-immunoglobulin M (IgM) was 1:160 and then azithromycin and methylprednisolone were applied for anti-inflammation. On 5th day, due to persistent fever, aggravated cough, and lesions on chest radiography progressed after conventional anti-infective therapy, the second FB was performed and found plastic bronchitis in the airway (Figure 2A). Bronchoalveolar lavage (BAL) fluid (BALF) was negative in other microorganism, except MP-DNA up to 5.1 × 10^8^ copies/ml. MP resistance mutation site 2063/2064 showed positive. No other etiological evidence was found in body fluid or secretions. On 9th day, the body temperature returned to normal level and the clinical symptoms and imaging improved.

**Figure 3:**
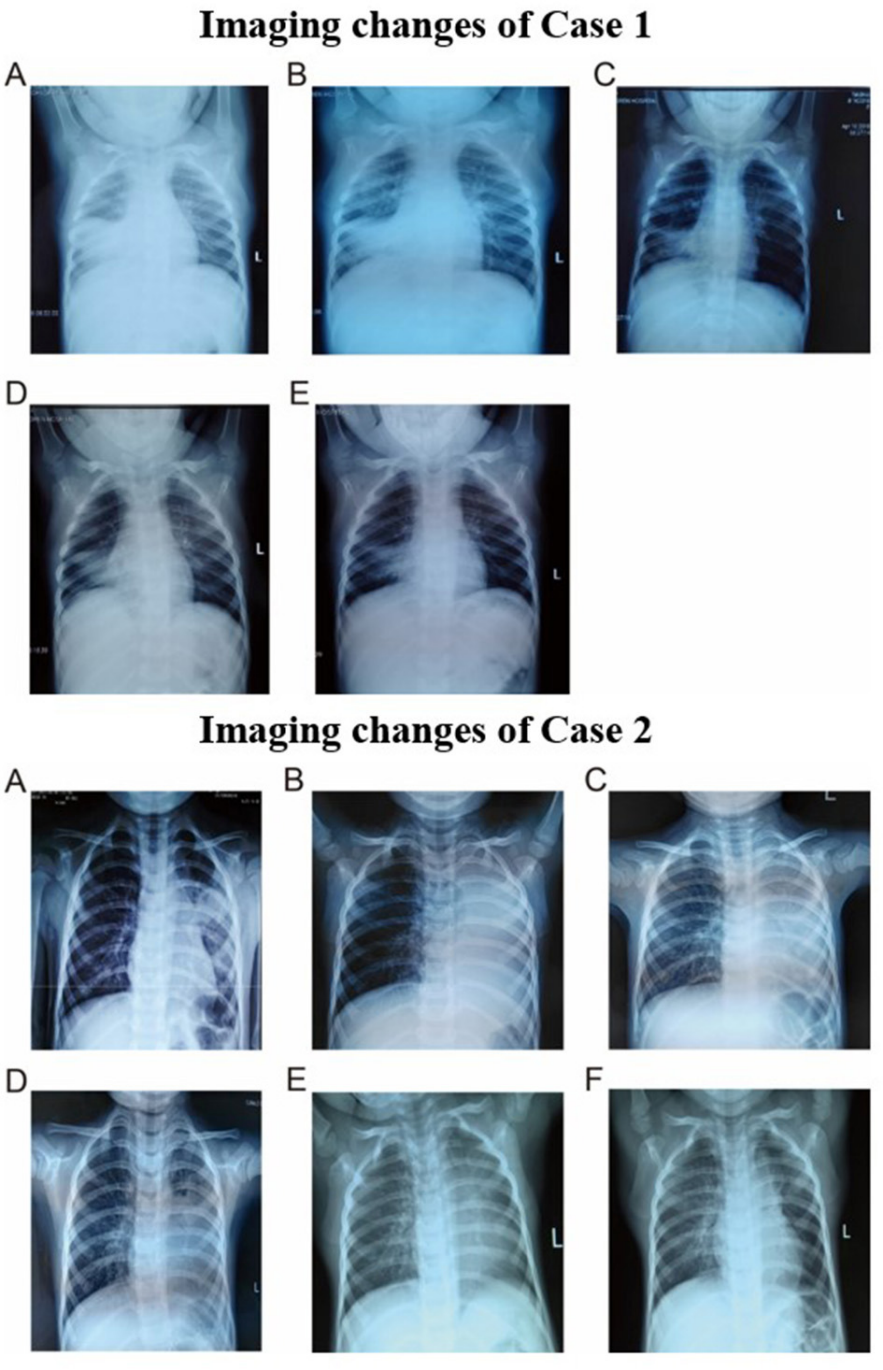
Imaging changes of case 1. **(A)** Chest radiography on day 5. **(B)** Chest radiography on day 9. **(C)** Chest radiography on day 13. **(D)** Chest radiography on day 27. **(E)** Chest radiography on day 30. Imaging changes of case 2. **(A)** Chest radiography on day 1. **(B)** Chest radiography on day 4. **(C)** Chest radiography on day 5. **(D)** Chest radiography on day 8. **(E)** Chest radiography on day 18. **(F)** Chest radiography on day 48.

**Figure 4:**
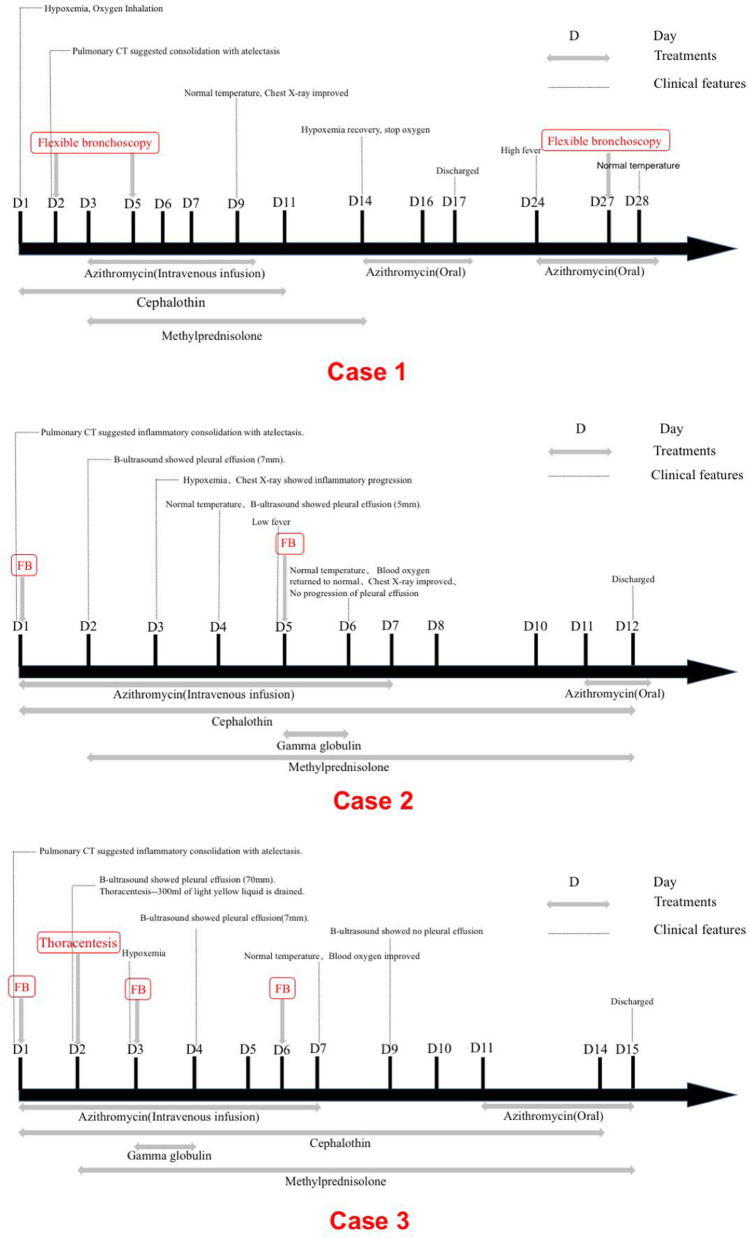
Timelines of disease course.

On 14th day, the blood oxygen turned to normal and stopped oxygen inhalation. The titer of MP-IgM was increased to 1:10240. She was discharged on the 17th day. However, on 24th day, she had sudden high fever. Because she was still not getting better after 3-day conventional anti-infective therapy and persistent consolidations in X-ray, the third FB was used on 27th day on outpatient department and removed bronchial casts. Her temperature was normal on the next day and the chest X-ray on the 30th day had great improvement (Figures 3, 4). She did not present any discomfort within 60 days.

### Case 2

A 2.4-year-old girl was hospitalized on October, 2015 due to 3-day persistent fever and cough, without underlying disease. Reservoir mask of 10 l/min was utilized for ventilatory support. Due to pulmonary CT suggested inflammatory consolidation with atelectasis, the first FB was used for treatment and etiological diagnosis. After 2 days of cephalothin and azithromycin treatment, she still had fever and cough and developed hypoxemia. Arterial blood gas revealed a pH of 7.44, PaCO_2_ of 45 mm Hg, PaO_2_ of 52 mm Hg, and oxygenation index of 247. Then, nasal high-flow oxygen of 10 l/min was adopted and methylprednisolone was applied for anti-inflammation. Physical examination revealed temperature 38.3°C, heart rate 135 beats/min, respiration 45 times/min, blood pressure 85/50 mm Hg, transcutaneous oxygen saturation 93% without oxygen administration, fatigue, flaring of nares, and decreased respiratory sound in the left lung. Cardiovascular, nervous system, extremities, ANA, and ENA examinations were normal. The routine blood tests indicated Hb 118 g/l, WBC 6.25 × 10^9^/L, N 55.3%, L 37.6%, and CRP 26 mg/l. On day 4, pulmonary CT suggested inflammatory consolidation accompanied with left pleural effusion (Figure 1B).

Fiberoptic bronchoscopy and pathological results revealed fibrinoid formation in the left upper lobe and lower lobe, indicating plastic bronchitis (Figure 2B). BAL fluid was negative in etiological, except for MP-DNA and Epstein Barr (EB)-DNA up to 1 × 10^8^ copies/ml and 4.8 × 10^5^ copies/ml, respectively. MP resistance mutation site 2063/2064 was also positive. No other etiological evidence was found in body fluid and secretions. Hypokalemia (K 3.28 mmol/l) and dysfunction of blood coagulation (Table 1) occurred during disease. On 5th day, chest radiography showed increased patchy shadows in the left lung (Figure 3). Therefore, the second PB was performed to relieve bronchial casts. After administration with methylprednisolone of 10 mg/kg/d and gamma globulin of 2 g/kg, body temperature returned to normal and anoxia was improved on the 6th day. The methylprednisolone was applied for 11 days. She was discharged on the 12th day and the titer of MP-IgM was increased by eight times on the 14th day (Figure 4). In the follow-up, she still had cough on the 18th day. On the 48th day, the chest X-ray turned to normal (Figure 3).

**Table 1:** General information of FMPP children.

**The basic characteristics**	**Case 1**	**Case 2**	**Case 3**
Age (year)	1.9	2.4	4.3
Gender	Female	Female	Male
Found acute lung injury (d)	4	5	7
Extrapulmonary complication	Myocardial and liver damage	Hypokalemia, coagulation dysfunction	Rash, liver damage, hyponatremia
WBC (× 10^9^/L; 4.0–10.0)	7.83	6.25	12.2
N (%; 45–77)	53.3	55.3	73.3
L %	40.4	37.6	27.1
CRP (mg/L; 0–8)	156	26	38.8
IL-6 (pg/mL; 0.00–7.00)	5.98	52.78	147.3
FER (ng/mL; 13.00–150.00)	32.21	182.9	4355
PCT (ng/ml; 0–0.05)	0.96	0.21	1.56
PT (sec; 10.0–16.0)	10.9	11.9	13.2
APTT (sec; 20.0–40.0)	28.2	27.9	36.1
Fg (g/L; 1.80–4.00)	2.855	4.415	2.567
D-dimer (mg/L; 0.00–0.55)	1.4	1.5	—
Cr (u mol/L; 23–37)	20	28	33
ALT (U/L; 7–40)	16	11	1595
AST (U/L; 13–35)	51	35	2031
LDH (U/L; 120–300)	449	551	2673
ALP (U/L; 142–335)	123	81	140
r-GT (U/L)	9	10	126
CK (U/L; 50–310)	338	77	110
CKMB (U/L; 0–24)	29	10	41
ESR (mm/h; 0–20)	30	22	4.0
Antibiotics after admission	Cephalothin, azithromycin	Cephalothin, azithromycin	Cephalothin, azithromycin
Anti-inflammatory therapy	Methylprednisolone 8 mg/kg/d	Methylprednisolone 10 mg/kg/d, Gamma globulin 2g/kg	Methylprednisolone 10 mg/kg/d
BAL fluid MP-DNA copies/ml	5.1 × 10^8^	1.0 × 10^8^	1.0 × 10^8^
Number of bronchoscopies	3	2	3
Average length of stay	17	12	15

*WBC, White blood cell; N, Neutrophils; L, Lymphocyte; CRP, C-reactive protein; IL-6, Interleukin-6; FER, Ferritin; PCT, Procalcitonin; PT, Prothrombin time; APTT, Activated partial thromboplastin time; Fg, Fibrinogen; ALT, Alanine transaminase; AST, Aspartate aminotransferase; LDH, Lactate dehydrogenase; ALP, Alkaline phosphatase; r-GT, r-glutamyl transpeptidase; CK, Creatine kinase; CKMB, creatine kinase-MB; ESR, Erythrocyte sedimentation rate; BAL, bronchoalveolar lavage*.

### Case 3

A 4.3-year-old boy was admitted on April, 2016 due to 5-day fever and cough. He was in good health and had never been to hospital. Two days before admission, pulmonary CT from other hospital suggested inflammatory consolidation with atelectasis. Therefore, the first FB was performed to etiological diagnosis and atelectasis treatment on admission. FB revealed fibrinoid formation in the right upper lobe. After 2 days of treatment of cephalothin and azithromycin, the condition worsened and hypoxemia developed. Arterial blood gas revealed PaCO_2_ 43 mm Hg, PaO_2_ 56 mm Hg, and oxygenation index 266. Then, reservoir mask of 6 l/min was adopted for ventilatory support. Physical examination revealed temperature 38.0°C, heart rate 135 beats/min, respiration 32 times/min, blood pressure 90/60 mm Hg, and transcutaneous oxygen saturation 90% without oxygen administration, fatigue, and decreased respiratory sound in the right lung. Cardiovascular, nervous system, extremities, ANA, and ENA examinations were normal. Routine blood tests showed Hb 132 g/l, WBC 12.2 × 10^9^/L, N 73.3%, and L 27.1%. The biochemical examination revealed a result of aspartate aminotransferase (AST) 2,031 U/L, alanine transaminase (ALT) 1,595 U/L, lactate dehydrogenase (LDH) 2,673 U/L, creatine kinase-MB (CK-MB) 41 U/L, triglyceride 1.33 mmol/l, procalcitonin (PCT) 1.56 ng/ml, CRP 38.8 mg/l, and ferritin 4,355 ng/ml. MP-DNA reaching 1 × 10^8^ copies/ml in BAL fluid and MP resistance mutation site 2063/2064 were positive and MP-DNA of hydrothorax was 3.2 × 10^5^ copies/ml.

On the 2nd day, pulmonary CT suggested substantial pulmonary consolidation in the upper and middle lobes of the right lung accompanied by significant effusion (Figure 1C). Due to persistent fever and significant elevation on blood inflammatory indicators, an additional methylprednisolone of 10 mg/kg/d was added for anti-inflammation and it was applied for 14 days. B-ultrasound showed that the maximum depth of pleural effusion was about 70 mm. Then, thoracentesis was performed and 300 ml of light yellow liquid was drained. Due to plastic casts in the airway and persistent consolidations in X-ray, FB was performed on the 3rd day and 6th day again. His temperature returned to normal and blood oxygen improved on 7th day. On 9th day, B-ultrasound showed no pleural effusion. The chest X-ray was normal and the titer of MP-IgM was increased by eight times on the 14th day. This patient had a fever for a total of 7 days after admission and discharged with azithromycin (oral) on the 15th day (Figure 4). No other etiological evidence was found in body fluid and secretions. She was followed-up without any discomfort on the 22nd and 52nd day.

## Discussion

*Mycoplasma pneumoniae* pneumonia has been reported in 10–40% of community-acquired pneumonia cases, younger children (<5 years of age) and school-aged children are prone to MP infection (6, 7). Cases of this study were also young children aged from 1.9 to 4.3 years old, suggesting that MP infection in this age group deserves attention. Our case 1 was initially suspected of bacterial infection due to obviously elevated CRP. In addition, because the patients were preschool children, we also considered whether these cases have virus infection, especially EB-DNA was found to be positive in BALF of case 2. We tested the virus [adenovirus, respiratory syncytial virus, influenza virus, EB virus (EBV), rhinovirus, and human metapneumovirus] and the culture of bacteria, fungi, and *Mycobacterium tuberculosis* through nasopharyngeal specimens and BALF. However, we found no other pathogens co-infection, except *Mycoplasma*, and the EBV antibodies in serum were all negative in case 2. Therefore, we believe that the etiology of our three cases of FMPP is only *Mycoplasma*.

Several studies have defined FMPP as MPP combined with hypoxemia, acute respiratory distress syndrome, or acute respiratory failure (3, 8, 9). Rapid progress of the disease is a prominent feature of FMPP. The shorter the course of disease, the more severe the disease, showing fulminant characteristics. In this case study, three cases also experienced rapid onset of hypoxemia, which was completely similar with previous reports. Key clinical findings of FMPP involve respiratory failure with diffuse consolidation or an abnormal interstitial pattern on a chest radiograph (3). The main CT manifestations of MPP include bronchial wall thickening, central lobule, ground-glass lesion, and inflammatory consolidation (10). However, the imaging manifestations of FMPP are lack of specificity, most of which are bidirectional diffuse inflammatory infiltration, accompanied by pleural effusion or large inflammatory consolidation (5). Besides, it may be accompanied by atelectasis and pneumothorax, which is similar to image performance of three cases reported in this case study.

It has been reported that a 15-year-old healthy child had sudden fever and respiratory failure, accompanied by headache, lethargy, difficult to wake up and then died of FMPP combined with hypoxic brain damage and brain edema on the next day (5). Miyashita et al. found that the average time from FMPP onset to respiratory failure was 6.5 days, with two cases only lasting 3 days. Of the 13 patients reported by Miyashita, 9 patients needed mechanical ventilation (11). Our three patients started with high fever and cough and rapidly progressed to respiratory failure within 7 days. Extrapulmonary manifestations included vomiting, diarrhea, convulsion, anorexia, weakness, and headache (12). Besides, liver dysfunction is a typical extrapulmonary complication of FMPP (12). FMPP can lead to the occurrence of hemophagocytic syndrome (13). Coagulation dysfunction occurs in patients with disseminated intravascular coagulation (14). In addition to acute lung injury, pleural involvement, skin rash, liver dysfunction, coagulation dysfunction, and electrolyte disturbance were also found in these cases, suggesting that FMPP was a part of systemic inflammation associated with multiple organ involvement in addition to pulmonary manifestations. The extrapulmonary complications of the three cases were different. In case 1, the girl developed myocardial and liver damage. However, in case 2, hypokalemia and coagulation dysfunction were occurred in the girl. In case 3, he developed rash, liver damage, and hyponatremia.

In addition to the degree of organ damage, the outcome of FMPP is related to means and intensity of intervention (5). Several studies have shown that bronchoscopy is helpful in the diagnosis and treatment of severe or refractory pneumonia in children, especially in children with atelectasis (15). When children developed recurrent pneumonia, suspected foreign body aspiration, and radiographic abnormalities (atelectasis and recurrent/persistent consolidations), we should be aware of the application of diagnostic FB (16, 17). The three cases in this study had severe pneumonia with atelectasis, which conformed to the application of diagnostic FB indications. Discharge contraindications and early application of FB can relieve airway obstruction and carry out etiological examination of alveolar lavage fluid. If the fever does not subside and the chest X-ray does not improve in 3–5 days after the first FB operation, the patient will receive multiple FB and BAL treatments (18). Bronchoscopy examination revealed the formation of plastic bronchial tree in the airway, impeding the ventilation function of alveolar tissue, and, thereby, leading to respiratory and circulatory failure and life-threatening complications. Therefore, timely bronchoscopy should be performed on treatment to remove inflammatory foreign bodies in the airway. In addition to the detection of bronchoplastic under direct vision, acting local lavage, and relieving airway obstruction, it is of great significance to ensure early diagnosis by MP-DNA examination of BALF (4, 19). In this case study, after taking out the plastic material with forceps, BAL was carried out with normal saline, followed by pathogen detection of the BALF. Then, antimicrobial and immunomodulatory therapy were performed after identifying the pathogen.

Although the mechanism of FMPP is largely unknown, growing evidences have confirmed that excessive host cell immune response plays a critical role in the progressionof FMPP (3). Early use of steroid is of prominent efficacy on the relief of symptoms and pulmonary imaging of FMPP (9). For patients with FMPP, especially those with severe extrapulmonary complications, treatment with glucocorticoid (GC) pulse can lead to rapid improvement in clinical symptoms (20). In case 1, due to the persistent high fever and severe clinical symptoms, she received an initial dose of methylprednisolone of 2 mg/kg/d, but still had fever. When the final dose was adjusted to 8 mg/kg/d, the temperature of the child returned to normal. Several studies have reported that some children with severe or refractory MP need methylprednisolone at doses ranging from 10 to 30 mg/kg/d (21, 22). It can be seen that conventional dose of GC therapy is ineffective in some cases, while high-dose GC therapy significantly downregulated the cell-mediated immune response (23, 24). In cases 2 and 3, due to the large amount of pleural effusion in the short term, we applied methylprednisolone for high-dose therapy in time. Therefore, the pleural effusion and clinical symptoms recovered rapidly. Our previous study found that high inflammatory index, lung consolidation, and pleural effusion may be predictors, which can guide the pulse dose of GC for the children with RMPP (25).

In addition, intravenous immunoglobulin, plasmapheresis, and hemofiltration have also been reported. Youn et al. have revealed that early application of immune-modulatory therapy (methylprednisolone and intravenous immunoglobulin) plus antimicrobial therapy may prevent disease exacerbations and reduce disease severity without associated side effects (26). Shen et al. have also proved the effectiveness of the combination of immunoglobulin and GC on the treatment of severe MPP children (27). In this case study, three cases received GCs based on adequate antimicrobial therapy and two of them received intravenous gamma globulin. When the body temperature of these children was normal for 24–48 h, the blood routine (WBC and N) and blood inflammatory indicators (CRP, LDH, and ferritin) were reduced.

This case study reports a group of younger MPP children characterized by rapid hypoxemia, acute lung injury, and multiple organ involvement. After active antimicrobial therapy, immunotherapy, GC, and pulmonary interventional therapy, these children recovered well. In summary, early identification and prompt intervention can save life of children and reduce sequelae. The main limitation of this case study was small sample size based on three cases, without matched controls. Further study is needed to expand the sample size and conduct in-depth study on intervention measures.

## Data Availability Statement

The original contributions presented in the study are included in the article/supplementary material, further inquiries can be directed to the corresponding authors.

## Ethics Statement

Authors obtained a written informed consent from the parents of the patient for the publication. A written informed consent was obtained from the minor(s) legal guardian/next of kin for the publication of any potentially identifiable images or data included in this article.

## Author Contributions

TZ contributed to the care for patients. WG contributed to the collecting data. TZ and CH contributed to the drafting the article. CH, WG, and JN contributed to the revising it for intellectual content. CC and YX contributed to the final approval of the completed article. All authors have read and approved the final manuscript.

## Conflict of Interest

The authors declare that the research was conducted in the absence of any commercial or financial relationships that could be construed as a potential conflict of interest.

## Publisher's Note

All claims expressed in this article are solely those of the authors and do not necessarily represent those of their affiliated organizations, or those of the publisher, the editors and the reviewers. Any product that may be evaluated in this article, or claim that may be made by its manufacturer, is not guaranteed or endorsed by the publisher.
